# Spatial and temporal analysis of myocardial infarction incidence in Zanjan province, Iran

**DOI:** 10.1186/s12889-021-11695-8

**Published:** 2021-09-14

**Authors:** Mohsen Soleimani, Nasser Bagheri

**Affiliations:** 1 Department of Information Technology, Zanjan University of medical sciences (ZUMS), Zanjan, Iran; 2 Center for Mental Health Research College of Health and Medicine, Australian National University, Canberra, Australian Capital Territory Australia

**Keywords:** Spatial analysis, Myocardial infarction, Incidence rate, Spatial autocorrelation, Hot spot analysis, Cluster and outlier analysis

## Abstract

**Background:**

Myocardial Infarction (MI) is a major important public health concern and has huge burden on health system across the world. This study aimed to explore the spatial and temporal analysis of the incidence of MI to identify potential clusters of the incidence of MI patterns across rural areas in Zanjan province, Iran.

**Materials & methods:**

This was a retrospective and geospatial analysis study of the incidence of MI data from nine hospitals during 2014–2018. Three different spatial analysis methods (Spatial autocorrelation, hot spot analysis and cluster and outlier analysis) were used to identify potential clusters and high-risk areas of the incidence of MI at the study area.

**Results:**

Three thousand eight hundred twenty patients were registered at Zanjan hospitals due to MI during 2014–2018. The overall age-adjusted incidence rate of MI was 343 cases per 100,000 person which was raised from 88 cases in 2014 to 114 cases in 2018 per 100,000 person-year (a 30% increase, *P* < 0.001). Golabar region had the highest age-adjusted incidence rate of MI (515 cases per 100,000 person). Five hot spots and one high-high cluster were detected using spatial analysis methods.

**Conclusion:**

This study showed that there is a great deal of spatial variations in the pattern of the incidence of MI in Zanjan province. The high incidence rate of MI in the study area compared to the national average, is a warning to local health authorities to determine the possible causes of disease incidence and potential drivers of high-risk areas. The spatial cluster analysis provides new evidence for policy-makers to design tailored interventions to reduce the incidence of MI and allocate health resource to unmet need areas.

**Supplementary Information:**

The online version contains supplementary material available at 10.1186/s12889-021-11695-8.

## Introduction

It is well known that cardiovascular diseases (CVD) are one of the most important public health concerns across the world [1]. Myocardial infarction (MI) is one of the most important CVD that can lead to serious complications and even death [2]. The incidence of MI, commonly known as a heart attack, has no geographic, socioeconomic, or gender boundaries [3]. The mortality rate of MI was 265 cases in the world, 224 cases in the Eastern Mediterranean, and 221 cases per 100,000 person in Iran [4, 5]. Majority of deaths due to MI can be prevented by controlling behavioral and environmental risk factors. Individual characteristics, lifestyle, and environmental factors play an important role in the incidence of MI [6–8]. Most of these risk factors may interact with each other and increase the incidence rate of MI. A study by Namayande in 2016, for example, reported a significant relationship between the incidence of MI and environmental factors such as air pollution and heavy metals like lead, mercury, selenium, cobalt, nickel [9].

Spatial analyses of the incidence of MI will generate new knowledge to identify high risk areas and investigate the potential impact of environmental risk factors on the incidence of MI [10–12]. Geographic Information Systems (GIS) is a powerful tool to support geocoding MI patients’ locations, conducting spatial analysis and visualizing the incidence of MI patterns at a finer geography level. GIS has the capacity to link spatial data with different source of MI attribute data to develop a big picture for the incidence of MI pattern across communities. This would enable policy-makers to identify the high risk areas of the incidence of MI and its potential associated risk factors [12, 13].

A number of studies examined the spatial distribution and the impact of environmental factors on MI incidence. A study by Liu used Moran’s I technique and hot spot analysis to explore the spatial pattern of MI and MI hot spots in older and low socioeconomics communities in Calgary, Canada from 2004 to 2013 [14]. Spatial analysis of the incidence of acute MI in Denmark showed that people who lived in MI clusters had a significantly lower economic status compared to the people who lived outside of the clusters [15]. Hot spot analysis of MI in an urban population in Yazd province, Iran showed a significant relationship between the proximity of MI clusters and industrial facilities, especially steel factories [9]. A study by Ahmadi in 2016 reported a specific pattern in the spatial distribution of MI among different provinces of Iran (Moran’s I: 0.75, *P* < 0.001) which was clustered in six areas of Iran included Semnan, Yazd, Kerman, North Khorasan, Mazandaran and Golestan provinces [16].

Although spatial analysis of MI was conducted in several countries across the world [10, 13–16], it has not yet been examined thoroughly at rural district levels (a finer geography level) and the majority of current studies were conducted at country scales [16]. Presently, there is little knowledge about the incidence rate of MI across rural areas. Zanjan province is an important industrial city with highly developed lead and zinc mines in Iran which many of them are located close to counties and rural areas. Spatial analysis of the incidence of MI generates new knowledge on spatial variations of the incidence of MI across communities at rural and urban areas and helps to identify unmet areas where the incidence of MI risk is greater [16]. Spatial analysis of the incidence of MI at finer geography level will enable policy makers to tailor prevention strategies to areas where the MI risk is greater. The aim of this study is threefold; 1) investigate spatial autocorrelation in the pattern of the age-adjusted incidence rate of MI 2) explore the potential significant clusters (hot or cold spots) and [3] visualize the spatial-temporal pattern of crude and age-adjusted incidence rate of MI at districts levels in rural areas of Zanjan province from 2014 to 2018.

## Materials & methods

### Data source

**Fig. 1:**
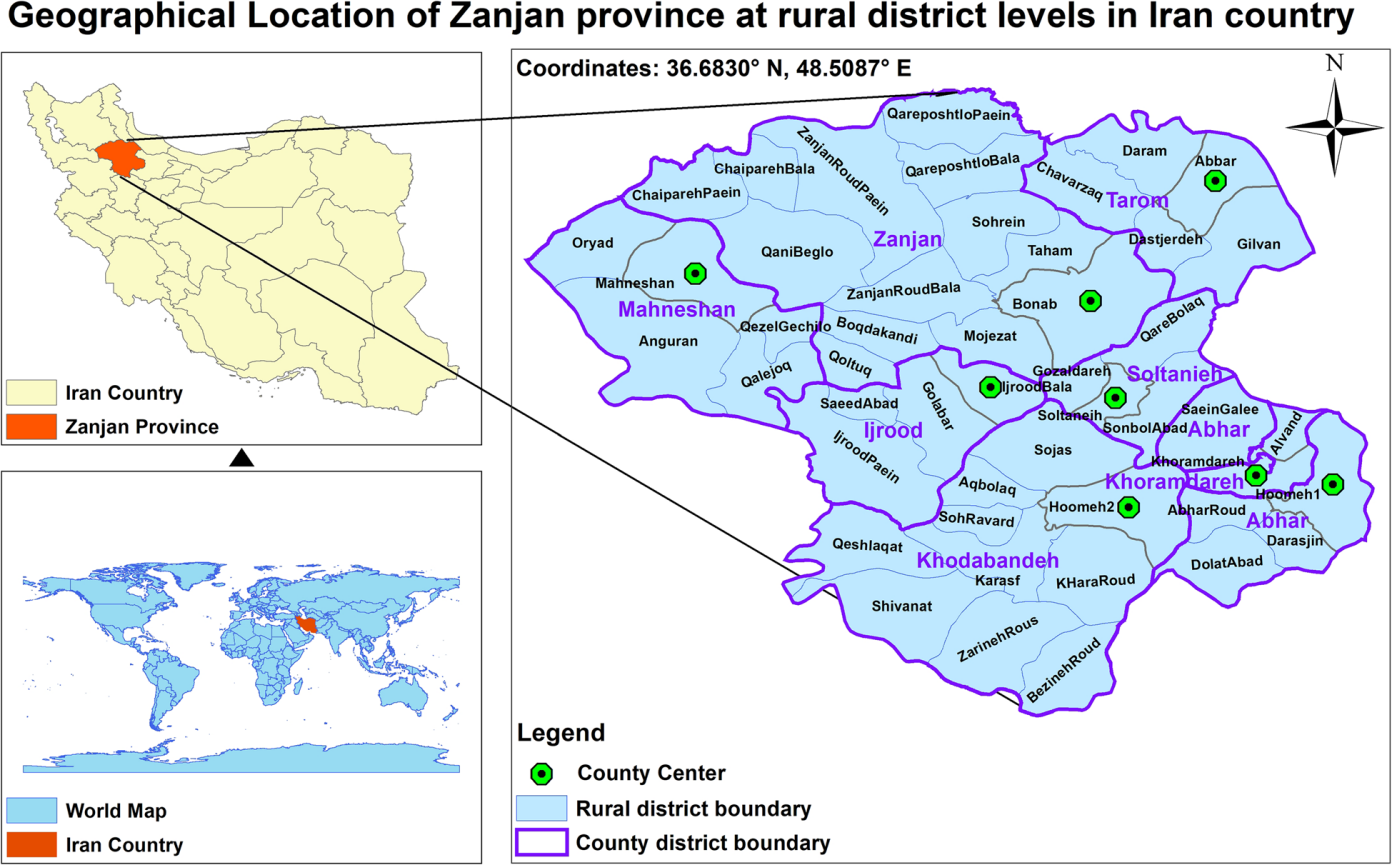
Geographical Location of rural districts in Zanjan province, Iran

This study was a cross-sectional and retrospective geospatial analysis of the incidence of MI at Zanjan province in 2021. Zanjan province is one of the 44 provinces of Iran located at the northwest which included 48 rural district and 8 counties with a population of 1,057,461 person and with an area of 21,773 Km^2^ (Fig. 1). Data were obtained from nine hospital information system (HIS) databases with attributes include sex, discharge diagnosis code, residence address, admission date and discharge date. HIS was implemented in some hospitals since 2014 and there was not complete data about the incidence of MI prior to 2014, therefore, we used data registered patient records in HIS databases over a five-year period (between March 21, 2014 and March 21, 2019). MI was defined using the International Classification of Diseases 10th revision (ICD-10) codes I21 and I22.

### Statistical analysis

**Fig. 2:**
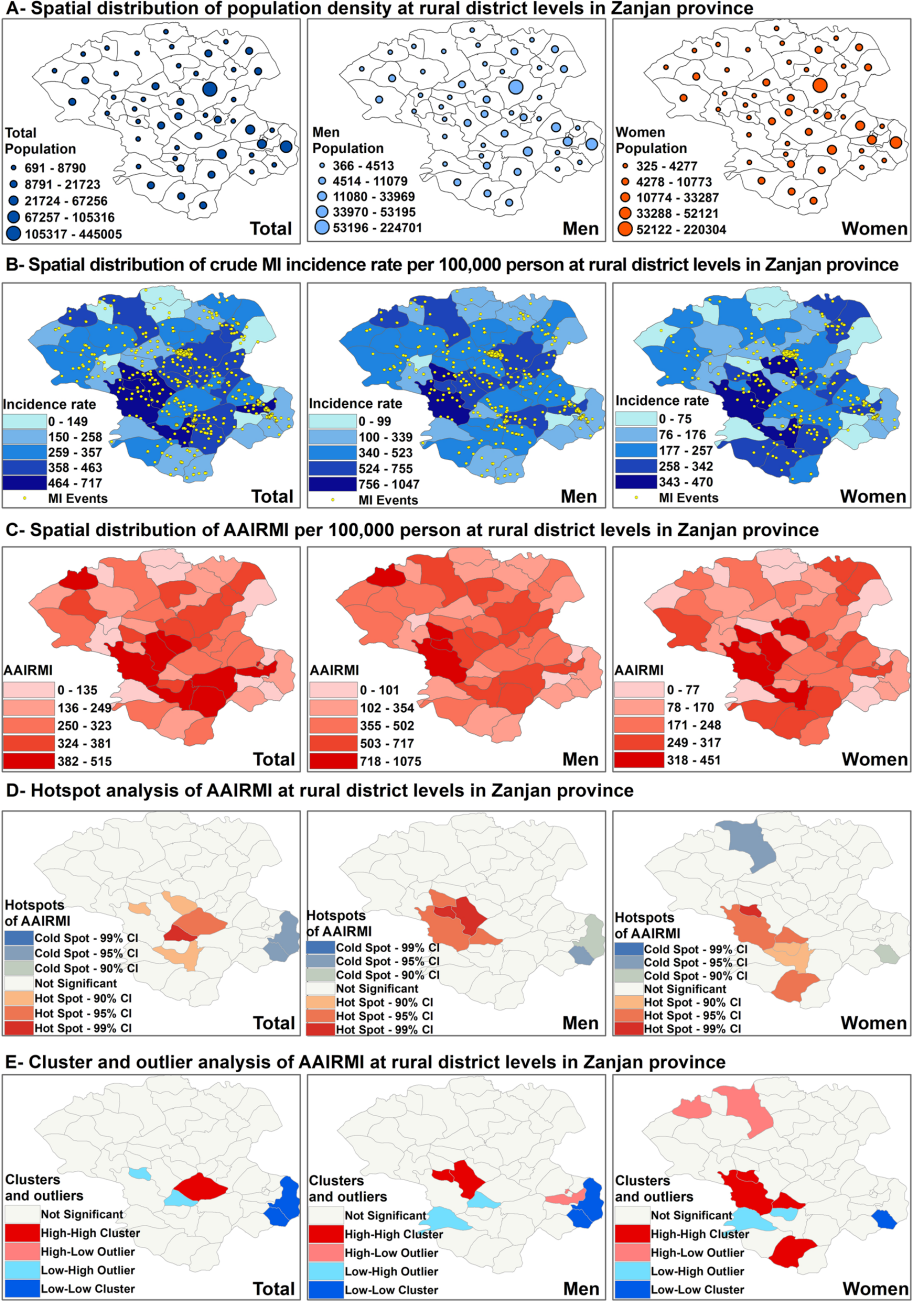
Spatial analysis of overall MI by gender at rural district level in Zanjan province

In Iran, the Government conducts a national census every 5 years. We used the 2016 census data to project the population of other years (2014, 2015, 2017 and 2018) by considering population’s annual growing rate reported by Statistical Center of Iran (Fig. 2A). Crude incidence rate of MI (CIRMI) and age-adjusted incidence rate of MI (AAIRMI) were calculated at province and rural areas at total, by genders and per years separately. The crude incidence rate of MI (CIRMI) was calculated for each rural district per 100,000 person by dividing the total number of MI cases in each area by its population and then multiplying by 100,000. Age adjusted rate allows fairer comparisons to be made between groups with different age distributions. A “standard” population distribution is used to adjust the MI rates. The age-adjusted rates are the rates that would have existed if the population under study had the same age distribution as the “standard” population. Therefore, they are summary measures adjusted for differences in age distributions. A direct standardization approach was used to calculate the age adjusted incidence rate of MI (AAIRMI) [17]. The mean population of Zanjan province from 2014 to 2018 was used to calculate the overall CIRMI and AAIRMI. The entire Iranian population in 2016 was used as the standard population to calculate the overall AAIRMI in Zanjan province. The entire population of Zanjan province for each year was used as the standard population to calculate the AAIRMI for each rural area and to calculate the AAIRMI among men and women on a yearly basis during the study period. We conducted spatial-temporal analysis of AAIRMI over years to determine the trend and high-risk areas of AAIRMI during 2014–2018. Temporal patterns of AAIRMI is very important factor for policy planning and can be useful in decision making on health resources allocation.

### Spatial analysis

Latitude and longitude coordinates of each patients’ residence address were obtained using google MyMap within a radius of half a kilometer. These data were exported to ARCGIS 10.7 software (ESRI Inc., Redlands, CA, USA) as a KML file for further spatial and statistical analyses.

Spatial analysis methods including spatial autocorrelation (Global Moran’s I), hot spot analysis and cluster and outlier analysis (Anselin Local Moran’s I) were used to examine potential clusters in the pattern of AAIRMI in the study area. We used AAIRMI as the input data for all spatial analysis to identify the spatial autocorrelation, hot spots and clusters of MI in Zanjan province. Identifying the spatial autocorrelation is essential before prejudicing and conducting any spatial clustering analysis [18]. Global Moran’s I analysis was conducted to investigate if there is any spatial autocorrelation in the pattern of MI. Given a set of features (rural district locations) and an associated attribute (age-adjusted incidence rate of MI values), Global Moran’s I tool determines whether AAIRMI pattern was clustered, dispersed, or random in Zanjan province, Iran. After identifying the spatial autocorrelation, it is crucial to identify the clusters and high-risk areas of MI using hot spots analyses techniques. There are several clustering analysis methods to determine the high-risk areas such as hot spot analysis, cluster and outlier analysis (Anselin Local Moran’s I), K-means, geographical weighted regression (GWR) [19]. Hot spot analysis and Anselin Local Moran’s I statistic were common spatial techniques to examine the clustering in the pattern of AAIRMI at Zanjan province [11, 16].

### Spatial autocorrelation (global Moran’s I)

Spatial autocorrelation is the natural tendency of a variable to represent similar values as a function of distance between the geographical locations at which it is being measured [20]. Strong autocorrelation occurs when there was a relationship between the values of a variable that are geographically close to each other [21]. Spatial autocorrelation analysis presents two graphically and numerically outputs. The Graphical output indicates the distribution pattern of data (scattered, clustered or a random pattern). The numerical output returns five values; Moran’s I Index, Expected Index, *P*-value, Z-score, and Variance. The Spatial autocorrelation tool is an inferential statistic, which means that the results of the analysis are always explained within the context of its null hypothesis [22]. The null hypothesis for the Global Moran’s I statistic in this study stated that AAIRMI was distributed randomly among rural district of Zanjan province during 2014–2018. When the *P*-value returned by Global Moran’s I statistic is statistically significant, the null hypothesis will be reject. If the value of Moran’s I index was close to + 1, AAIRMI distributed spatially clustered and if it was close to − 1, it was spatially scattered. Zero value of Moran’s I index show that AAIRMI distributed randomly (Additional file 1).

### Local indicators of spatial autocorrelation – hot spot (cluster) and outlier analysis

Global methods are more sensitive to departures from the null hypothesis [23]. Although they could recognize spatial structures, they are not able to identify the situation of clusters and where the clusters of high or low AAIRMI might occur. Local indicators of spatial autocorrelation (LISA) is one the hot spots analyses methods that identifies statistically significant hot spots (high values) and cold spots (low values) using the Getis-Ord Gi statistic [24–26]. Hot spot analysis was used to determine the clusters of rural district with high or low values of AAIRMI. An area considered as a hot spot when the area with high AAIRMI surrounded by a cluster of high AAIRMI and an area considered as a cold spot when the area with low AAIRMI surrounded by a cluster of low AAIRMI. Hot spot analysis returns an output feature class with a *P*-value, Z-score, and confidence interval (CI) bin field (Gi-Bin) for each feature in the input feature class. Features in the ±3 bins reflect statistical significance with a 99% CI; features in the ±2 bins reflect a 95% CI; features in the ±1 bins reflect a 90% CI; and the clustering for features in bin 0 is not statistically significant (Additional file 1).

Additionally, Anselin local Moran’s I is a type of local cluster detection method which was used to examine the spatial outlier of AAIRMI in this study. This tool determined spatial clusters of rural districts with high or low AAIRMI as well as spatial outliers. Spatial outliers refer to areas with values of AAIRMI that were discrepant from the neighboring regions. Anselin local Moran’s I was used to verify and complement the hot spot analysis, because it allows to detect areas where anomalies exists. The results of Anselin local Moran’s I showed aspects that may had been overlooked in hot spot analysis, while were interesting highlights, especially in those areas where different types of groupings coexist. The outputs of Anselin local Moran’s I classified areas to five groups included: not part of a cluster (or no significant cluster), High-high cluster (HH), High-low outlier (HL), Low-High outlier (LH) and Low-Low cluster (HH) [24] (Additional file 1).

### Conceptualization of spatial relationships

The conceptualization of spatial relationships (CSR) determining how features within a layer interact and influence each other. The influence is determined by either a distance relative to the target feature, or by sharing a boundary. CSR tells computers how to identify the neighbors around each individual feature and which features are eligible as neighbors with each particular feature. Polygons shape have a variety of irregularly size features and because their size and shape vary distances isn’t quite as straightforward as it is with points. There are several options for CSR included inverse distance, inverse distance squared, fixed distance band, zone of indifference, contiguity edges only, contiguity edges corners, get spatial weights from file, and it’s depending on what is being measured. One of the typically ways that deal with polygons is to work with contiguity rather than looking at distance. Contiguity says that polygons that touch one another call it qualify as neighbors, so when the analysis is done, any polygons that touched are going to be counted. Therefore, contiguity edges corners technique was used as conceptualization of spatial relationship in this study. In contiguity edges corners technique, polygon features that share a boundary, share a node, or overlap will influence computations for the target polygon feature.

Some polygons are going to have more neighbors than others. The number of neighbors is a likely to be a function of the size of polygon so the smaller polygons have more neighbors and polygons had located at the edge of the study area have fewer neighbors. Row standardization was used in this study to create proportional weights in cases where features had an unequal number of neighbors.

## Results

Sixty-eight thousand eight hundred forty-three patients with cardiovascular condition were hospitalized in Zanjan hospitals between 2014 and 2018 in Zanjan. We excluded 5238 cases because of incomplete or duplicated cases and ultimately we had 3820 cases with MI (Fig. 2B). The overall AAIRMI was 343 cases per 100,000 person which was higher in men compared to women (504 cases per 100,000 man vs 204 cases per 100,000 woman, *P* < 0.0001) (Fig. 2B). The highest overall AAIRMI was observed at Golabar, Khoramdareh and Hoomeh2 (515 cases, 506 cases and 504 cases per 100,000 person, respectively). IjroodPaein, Qoltuq and SaeedAbad had the highest AAIRMI among men (1075, 823 and 774 cases per 100,000 man, respectively), while Karasf, Golabar and Mojezat had the highest AAIRMI among women (451, 398 and 383 cases per 100,000 woman, respectively) (Fig. 2C).

Although Global Moran’s I statistic results was not significant at a significance level of 0.05 and the spatial distribution of overall AAIRMI was random in Zanjan (Moran’s Index: − 0.020779), hot spot analysis detected five hot spots of AAIRMI in the study area included AqBolagh with a 99% CI, Sojas with a 95% CI and Karasf, SaeedAbad and IjroodPaein with a 90% CI. It also determined two cold spots of AAIRMI at Hoomeh1 and Darasjin with a 95% CI. The spatial pattern of hot spots and cold spots was different among men compared to women. SaeedAbad, Golabar, IjroodPaein, Qoltuq and Aqbolag were determined as hot spots of AAIRMI in male patients and Darasjin and Hoomeh1 were identified as cold spots of AAIRMI, while in women, SaeedAbad, IjroodPaein, AqBolaq, Sohravard, Karasf and ZarinehRood were recognized as hot spots of AAIRMI and ZanjanRoudPaein and Darasjin were determined as cold spots of AAIRMI (Fig. 2D).

The results of Anselin Local Moran’s I analysis showed that there was one HH cluster of AAIRMI at center of the study area (Sojas) and two LL clusters of AAIRMI also detected at Hoomeh1 and Darasjin which was consistent with the results of hot spot analysis. SaeedAbad and Aqbulaq were determined as a LH outlier of AAIRMI. Figure 2 also shows clusters and outliers of AAIRMI by geneder. SaeedAbad and Golabar were detected as HH clusters of AAIRMI among men, while SaaedAbad, IjroodePaein, Aqbolaq and ZarineRood were determined as HH clusters of AAIRMI among women. Khoramdareh was determined as a HL outlier of AAIRMI among men in Anselin Local Moran’s I analysis, which was detect previously as the second high risk area of AAIRMI with 506 cases per 100,000 person. ZanjanroadPaein and ChaiparePaein were identified as a HL outlier of AAIRMI among women, which means these areas had a high AAIRMI and were surrounded with low AAIRMI neighbors. Sohravard and Qeshlaqat were determined a LH outlier of AAIRMI among both men and women which mean this areas had a low AAIRMI and were surrounded with high AAIRMI neighbors (Fig. 2E).

**Fig. 3:**
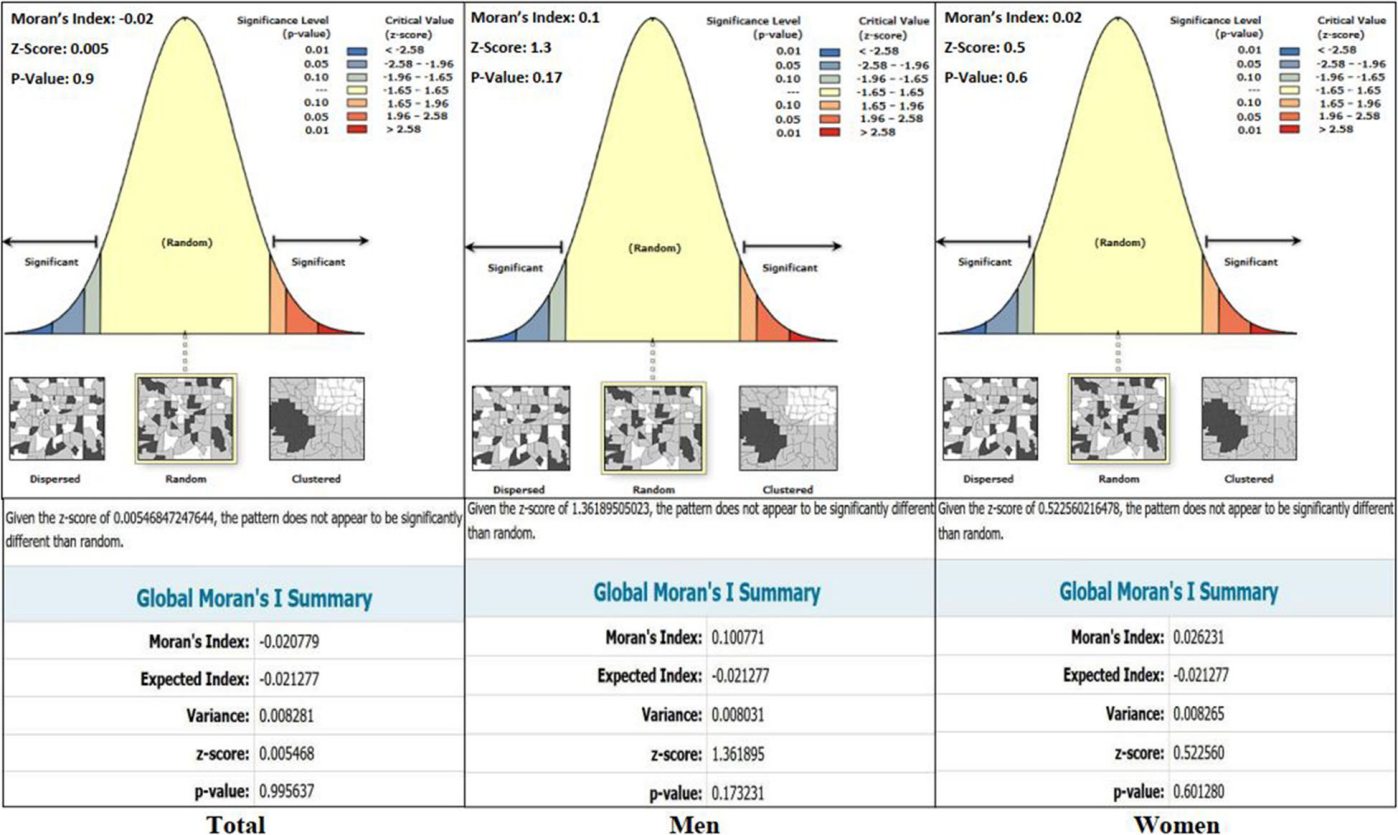
Graphical and Numerical outputs of Global Moran’s I for AAIRMI by gender in Zanjan

According to the graphical and numerical outputs of Global Moran’ I statistic and given the z-score of 0.005468, the spatial distribution of overall AAIRMI did not appear to be significantly different than random pattern at district levels in rural areas in Zanjan (Moran’s Index: − 0.020779) (Fig. 3). The spatial pattern of AAIRMI also can be regarded to be Random among men and women in the study area (Moran’s Index: 00.100771 in men, 0.026231 in women, respectively) (Fig. 3).

**Fig. 4:**
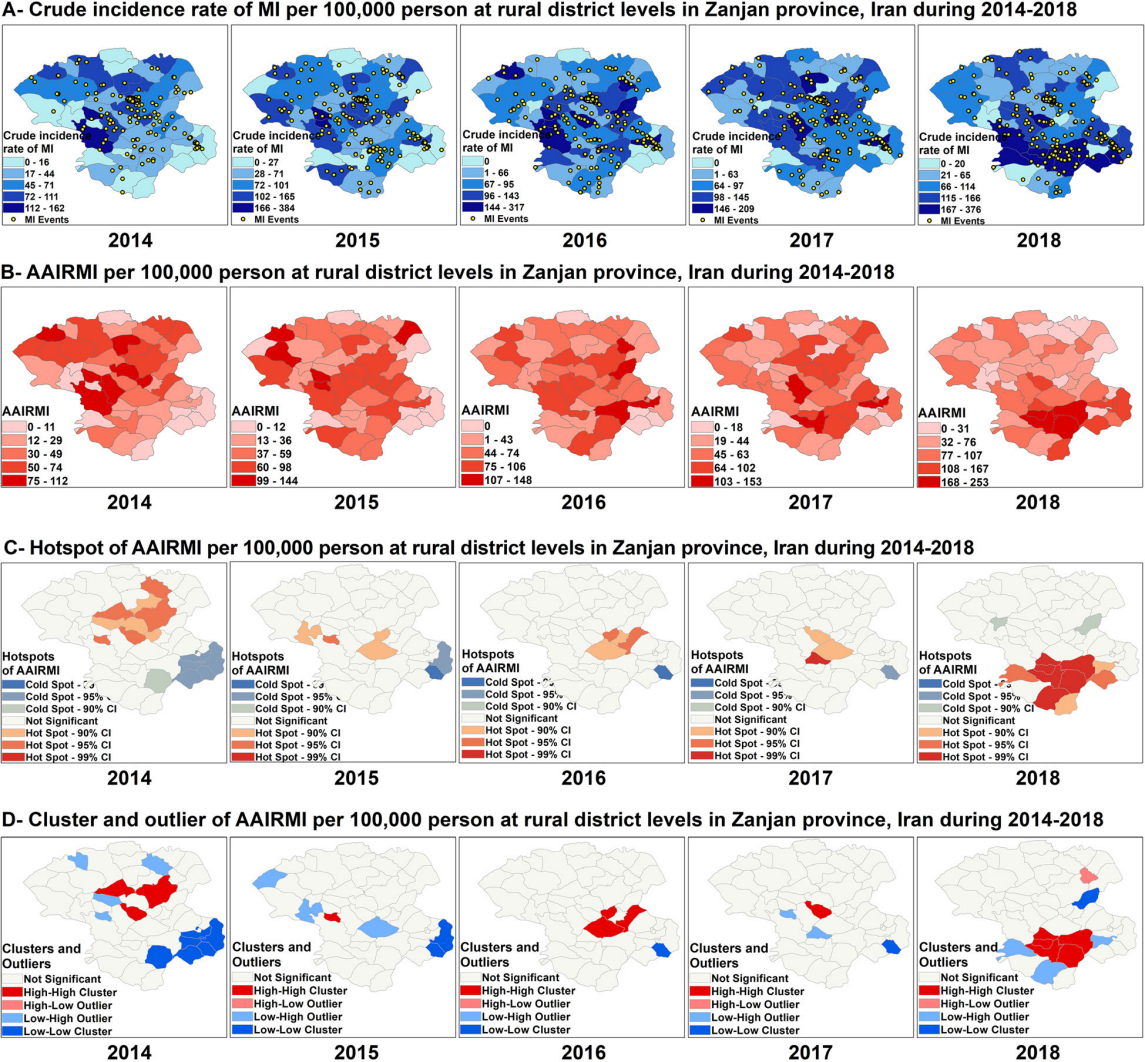
Spatial analysis of overall MI over time in Zanjan province from 2014 to 2018

The overall CRIMI and AAIRMI was different across the years and an ascending trend was observed in Zanjan during 2014–2018 (*P* < 0001) (Fig. 4A). The overall AAIRMI was increased from 88 cases in 2014 to 114 cases in 2018 per 100,000 person-year (a 30% increase). The mean of AAIRMI was 81 cases per 100,000 person-year over a five-year period in Zanjan (Fig. 4C). While the overall AAIRMI was decreased at Bonab as the most populated region in rural Zanjan (from 71 cases in 2014 to 69 cases in 2018 per 100,000 person-year), the highest increase of overall AAIRMI was occurred at Karasf which increased from 35 cases in 2014 to 253 cases in 2018 per 100,000 person-year, a 620% increase. A significant increase of overall AAIRMI also was observed at DolatAbad (from 0 to 167 cases), Sohravard (from 35 to 253 cases), Khararood (from 29 to 194 cases) and Hoomeh2 (from 24 to 189 cases) (Fig. 4B).

Spatial autocorrelation of overall AAIRMI showed that the Moran’s I statistic was significant in 2014 and 2018, (Moran’s Index: 0.132095 in 2014 and 0.295579 in 2018, respectively) and the spatial pattern was clustered only in these years (Table 1). As Fig. 4 reveals, the spatial distribution of hot spots and cold spots of AAIRMI was changed over a 5 years in Zanjan and shifted the south from the center of the study area. Ten hot spots and two cold spots were detected in 2018, which many of them were different from those were observed previously (Fig. 4C). Anselin local Moran’s I analysis showed that the spatial clusters of overall AAIRMI were different across the years during 2014–2018. The number of HH clusters was increased from three clusters in 2014 to five clusters in 2018, while the number of LL clusters was decreased from six clusters to one cluster during 2014–2018. One LL cluster of AAIRMI was observed at QaraBolaq in 2018 which was located at the east, and it was different from those were observed previously (Fig. 4D).

**Table 1:** Numerical outputs of Global Moran’s I for AAIRMI in Zanjan province from 2014 to 2018

	Year	Moran’s index	Expected index	Variance	Z-score	*P*-value	Pattern
Total	2014	0.132095	−0.021277	0.008322	1.681216	0.092721	Clustered
	2015	−0.036007	− 0.021277	0.008293	−0.161750	0.871502	Random
	2016	−0.010825	− 0.021277	0.008271	0.114916	0.908511	Random
	2017	−0.082883	− 0.021277	0.008082	−0.685277	0.493169	Random
	2018	0.295579	−0.021277	0.008038	3.534136	0.000409	Clustered
Men	2014	0.171553	−0.021277	0.008041	2.150404	0.031523	Clustered
	2015	−0.045255	−0.021277	0.007131	−0.283957	0.776443	Random
	2016	−0.001827	−0.021277	0.008252	0.214108	0.830463	Random
	2017	−0.117834	−0.021277	0.008144	−1.069968	0.284634	Random
	2018	0.174963	−0.021277	0.008124	2.177200	0.029466	Clustered
Women	2014	0.189901	−0.021277	0.008187	2.333896	0.019601	Clustered
	2015	0.006763	−0.021277	0.007901	0.315442	0.752426	Random
	2016	−0.062129	−0.021277	0.008261	−0.449474	0.653090	Random
	2017	0.040164	−0.021277	0.007677	0.701237	0.483155	Random
	2018	0.239113	−0.021277	0.007787	2.950813	0.003169	Clustered

**Fig. 5:**
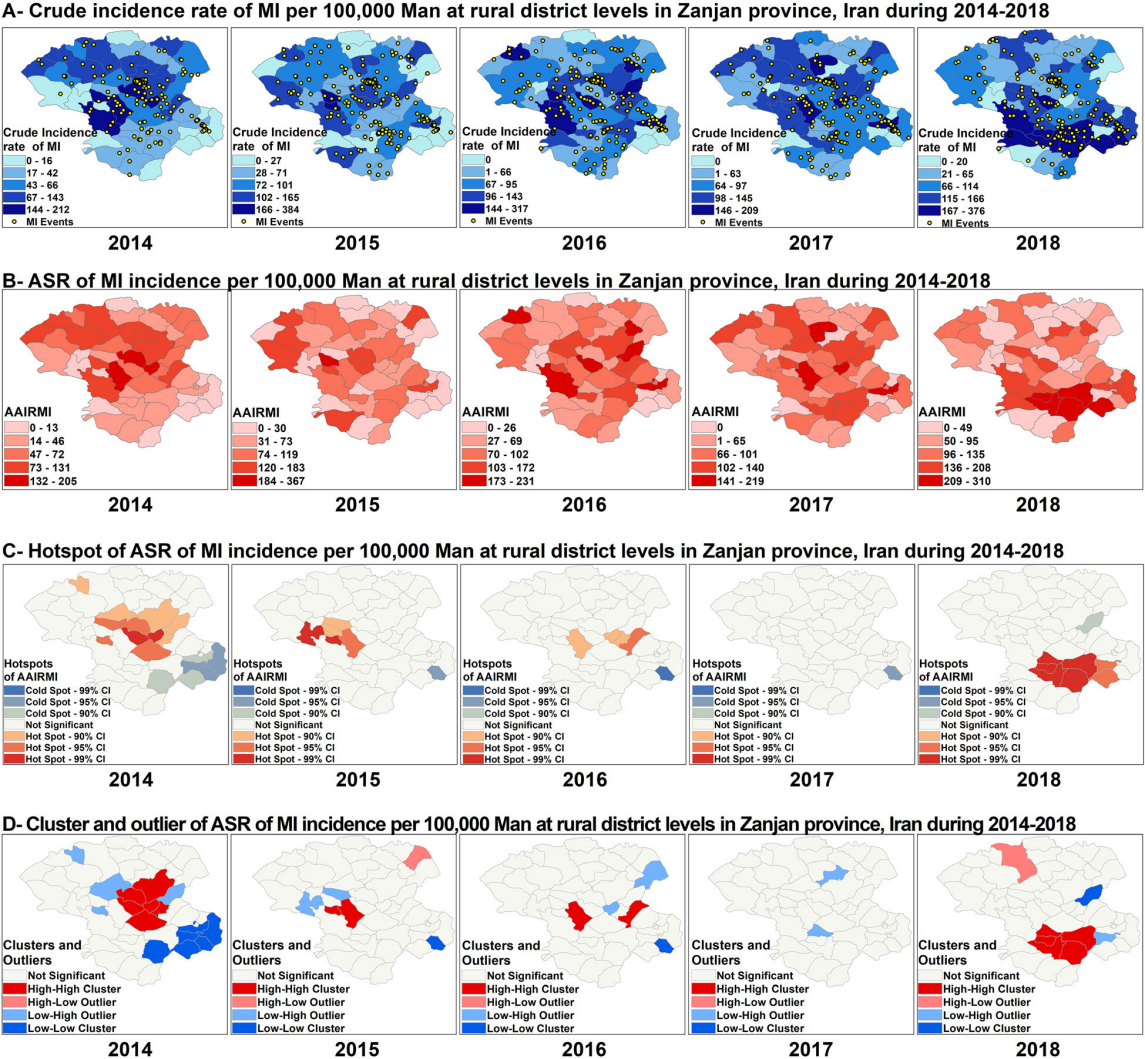
Spatial analysis of MI among Men in Zanjan province from 2014 to 2018

CIRMI and AAIRMI was not consistence among men across different years and an ascending trend was observed during 2014–2018 (*P* < 0001) (Fig. 5A). AAIRMI was raised from 70 cases in 2014 to 128 cases in 2018 per 100,000 man-year, an 80% increase. Among men, the highest overall AAIRMI was observed at IjroodPaein with 1046 cases per 100,000 man. The highest increase of AAIRMI in men was detected at DolatAbad which increased from 0 case in 2014 to 289 cases in 2018 per 100,000 man-year, while the major decline was observed in Gozaldareh which decreased from 205 cases in 2014 to 0 case in 2018. A significant increase of AAIRMI also observed among men at Sohravard (form 35 to 310 cases), Hoomeh2 (from 42 to 305 cases) and Karasf (from 32 to 257 cases) during 2014–2018 (Fig. 5B).

Spatial autocorrelation analysis showed that the Moran’s I statistic was significant among men only in 2014 and 2018 (Moran’s Index: 0.171553 in 2014, 0.174963 in 2018, respectively), and the spatial pattern of AAIRMI was clustered among men only in these years (Table 1). As Fig. 5 reveals, the spatial distribution of hot spots and cold spots of AAIRMI was changed among men over a 5 years in Zanjan, and the pattern shifted the south from the center. Among men, eight hot spots and one cold spot were recognized in 2018, which were different from those observed previously. The number of hot spots and cold spots were decreased from 11 hot spots and seven cold spots in 2014 to seven hot spots and one cold spot in 2018 (Fig. 5C). Anselin local Moran’s I analysis showed that the spatial clusters of AAIRMI was different among men across various years which was consistent with the results of hot spots analysis. Five HH clusters and one LL cluster was observed among men in 2018 which were different from those were observed previously. As Fig. 5 shows, the spatial distribution of clusters was changed over 2014 to 2018 and the spatial distribution of HH clusters was shifted the south from the center during 2014–2018 (Fig. 5D).

**Fig. 6:**
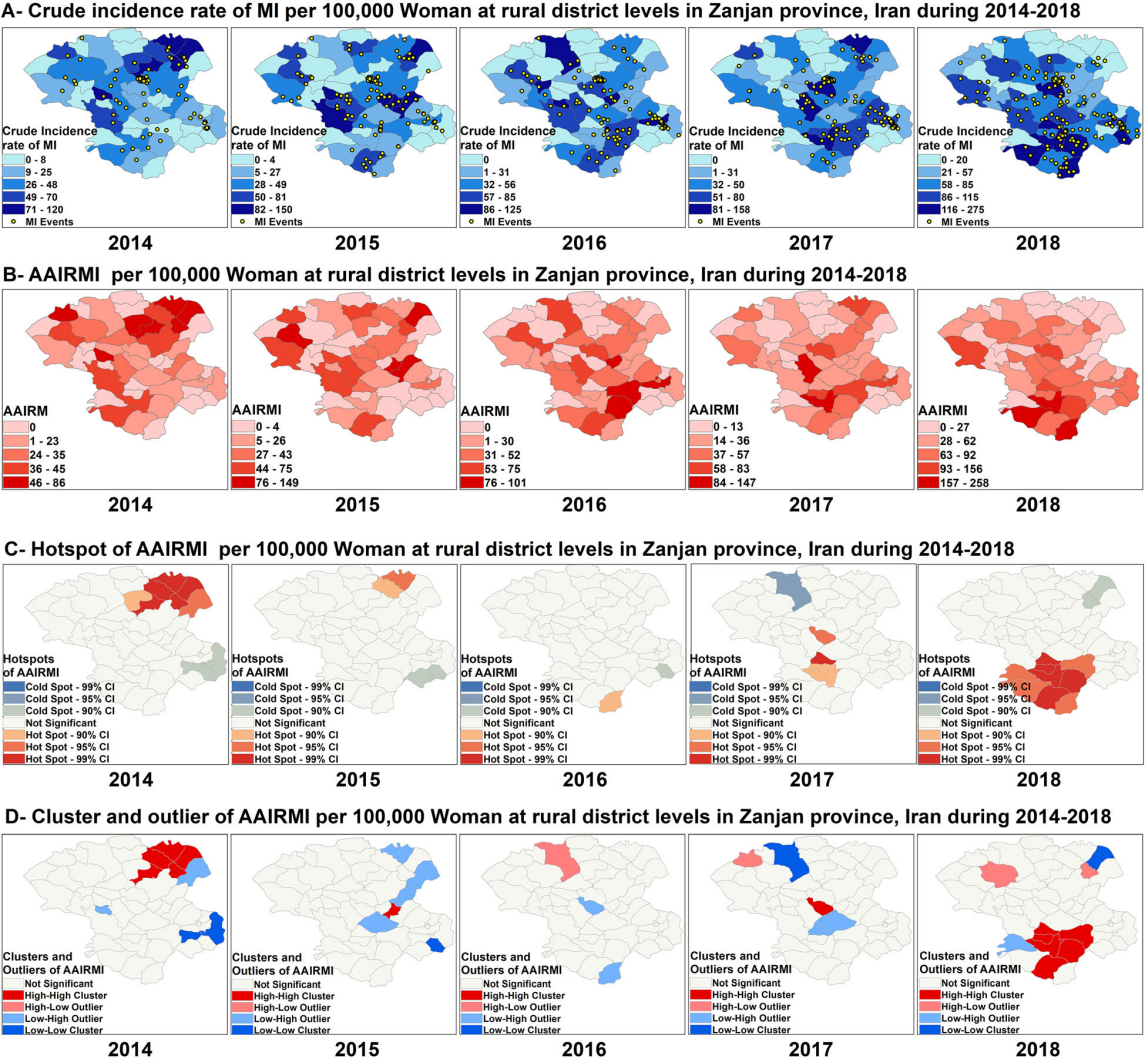
Spatial analysis of MI among Women in Zanjan province from 2014 to 2018

CIRMI and AAIRMI did not show a homogenous pattern in women across different years and presented an ascending trend from 2014 to 2018 (*P* < 0001) (Fig. 6-A). AAIRMI was increased among women from 27 cases in 2014 to 59 cases in 2018 per 100,000 woman-year representing a 120% increase. Among women, Karasf had the highest overall AAIRMI with 451 cases per 100,000 woman and also had the highest increase of AAIRMI compared to other regions, which increased from 37 cases in 2014 to 258 cases in 2018 per 100,000 woman-year, representing a 600% increase. A significant ascending trend also observed at BezinehRood region which increased from 0 case in 2014 to 182 cases in 2018 per 100,000 woman-year (Fig. 6B).

Although, Moran’s I statistic was significant among women only in 2014 and in 2018 (Moran’s Index: 0.189901 in 2014, 0.239113 in 2018, respectively), the spatial pattern of AAIRMI was random among women across other years (Table 1). As Fig. 6 shows, the spatial distribution of hot spots and cold spots of AAIRMI was changed among women over a 5 years period which shifted the south and the southwest from the northeast in the study area. Among women, nine hot spots and two cold spots were detected in 2018 which were different from those were observed previously. The number of hot spots was increased from seven hot spots in 2014 to nine hot spots in 2018, while the number of cold spots was decreased from three cold spots in 2014 to two cold spots in 2018 (Fig. 6C). Anselin local Moran’s I analysis showed that the spatial clusters of AAIRMI were different among women across various years, which consistent with the results of hot spots analysis. Six HH clusters located at the south, were observed among women in 2018 which were different from those observed in previous years. One LL cluster of AAIRMI was detected among women in Gilvan located at the northeast in 2018, while this region was determined as a HH cluster of AAIRMI in 2014 (Fig. 6D).

## Discussion

There is a limited research report on spatial analysis of MI compared to other diseases such as cancers in the world. In addition, most studies examined the spatial analysis of MI at country scales not at a finer geographical level. To the best of our knowledge this was the first study conducted to investigate the spatial analysis of AAIRMI at district levels in rural areas to determine the geographical distribution and high-risk areas of the incidence of MI in Zanjan province, Iran.

The overall AAIRMI was 343 cases per 100,000 person, and the mean AAIRMI per year was 81 cases per 100,000 person-year over a five-year period which was higher in men (68%) compared to women (28%). AAIRMI was higher in Zanjan compared to the national average (74 cases per 100,000 person-year) and it was lower than AAIRMI in other provinces of Iran such as Kerman (149 cases), North Khorasan (152 cases) and Semnan (132 cases per 100,000 person-year) [2]. The crude incidence rate of MI was 783 per 100,000 person-year in Yazd province, as one of the important industrial city in Iran, which was significantly higher than the results of present study [9]. The incidence rate of MI in Zanjan was different from neighboring provinces. It was 108 cases in Ardabil province (the north) [27], 39 cases in East Azerbaijan province (the west) [28], 97 cases in Qazvin province (the east) [29], 96 cases in Gilan province (the northeast) and 58 cases in Hamedan province (the south) [28].

According to the report of Statistical Center of Iran, Bonab, Hoomeh2 and Khoramdareh had the highest population density in Zanjan in 2016. (445,018, 105,330 and 67,260 persons, respectively) Bonab, the first metropolis and populated region which located at the center, classified as the 12th high-risk area of AAIRMI in this study with 370 cases per 100,000 person. In comparison to populated areas, while the population density of Golabar was lower (16,588 person), the highest overall AAIRMI was observed in this region and ranked the first high-risk area of AAIRMI. IjroodPaein and Qoltuq were defined as the high risk areas of AAIRMI among men (1075 cases and 823 cases per 100,000 man, respectively), while Karasf and Golabar were detected as the high-risk areas of AAIRMI among women. (451 cases and 398 cases per 100,000 woman, respectively).

Although the highest AAIRMI was observed in Golabar region, it didn’t have the highest population density compared to other regions in Zanjan. Therefore, besides population density, there should be other risk factors impacting the incidence of MI in this region. According to Rathore’s study, the main risk factors of MI include individual characteristics (family history, age and sex), lifestyle factors (physical activity, smoking and diet), underlying factors (socioeconomic factors and access to health care) and environmental factors (climate, temperature, humidity) [8].

According to Ahmadi’s study in 2014 [28] and the results of present study, AAIRMI was almost tripled in Zanjan over the past six yearss, which increased from 40 cases in 2012 to 114 cases in 2018 per 100,000 person-year, an 185% increase, while the population density increased only 4%. The highest overall AAIRMI was observed at Karasf region which increased from 35 cases in 2014 to 253 cases in 2018 per 100,000 person-year, a 600% increase. An ascending trend of The incidence of MI was reported in Iran during 1992 to 2004, which was similar to the trend of The incidence of MI in developing countries and consistent with present study [2]. The trend of the incidence of MI was different in developed countries compared to developing countries. Developed countries such as Japan [30], Korea [31], European countries [5] and the United States [32] experienced a descending trend of The incidence of MI in recent years which was in contrast. Several reasons explain this decline such as controlling CVD risk factors, smoking cessation programs, lifestyle changes, quick use of percutaneous coronary angioplasty, use of drug eluting stents and evidence-based medications in the prevention of major adverse of MI events [2, 30–32].

Spatial analysis plays an important role to visualize the spatial distribution of disease and identify the potential high-risk areas of the incidence of MI [2]. Hot spot analysis and Anselin Local Moran’s I are advanced statistical techniques, which were utilized in a number of research in health context to explore the potential significant clusters of disease events [21, 24, 33, 34]. When mapping the spatial distribution of AAIRMI using color coding techniques to classify the severity of incidence among areas, different results can be obtained by changing the classification range of incidence severity. Therefore, this approach cannot be useful to detect the real and potential high or low risk areas of AAIRMI alone and it might misdirect the results [18]. Global Morans’ I identifies spatial autocorrelation AAIRMI pattern. This techniques determines whether MI pattern is random, clustered or scattered, but it cannot specify where clusters are located [21]. Hot spot and Anselin Local Moran analysis be able to recognize the location of clusters and identify the spatial variation of AAIRMI [24]. Therefore, we need to run the global Moran’ I to investigate if the MI incident is a spatially auto correlated or not before conducting a hot spots analysis.

AAIRMI did not show a homogeneous pattern across rural region of Zanjan province during 2014–2018(*P* < 0.0001). The results of studies conducted in Ardabil [27] and Yazd [9] provinces as well as the studies conducted in Denmark [15], Sweden [13], Canada [14], and United State [32] also showed a different spatial distribution of The incidence of MI among various areas which were in line with our findings. Spatial autocorrelation showed that the spatial distribution of overall AAIRMI was random and there were different hot spots and clusters of AAIRMI in the study area. Various studies also showed that the incidence of MI was clustered among different geographical areas and support the efficiency of geo-spatial analyses to identify the potential high-risk areas of MI which consistent [3, 9, 10, 16].

One hot spot with a 99% CI, one hot spot with a 95% CI and three hot spot with a 90% CI were detected in the neighboring region of Golabar. This study was consistent with the results of a study conducted in Ardabil province, which identified hotpots of The incidence of MI using hot spot analysis [27]. The majority of clusters and outliers of AAIRMI were detected in rural regions of Zanjan province. Anselin Local Moran’s I method was applied in different studies to determine the cluster of MI among various areas. For example, Khalkhal County was recognized as a LH outlier of MI in the first 3 years of the study, while no clusters was defined among the counties of Ardabil province [27]. The results of studies conducted in Iran [16], Canada [14], Denmark [15], USA [11], Sub-Saharan Africa [22], Korea [31] and Sweden [13] support the efficiency of Anselin Local Moran I analyses to identify the potential high-risk areas of diseases and their results are in line with our findings. The results of this study showed that the highest AAIRMI was observed in rural areas compared to urban areas. Majority of people living in rural areas are farmer and related to agriculture work and have a poor socio-economic status. Lifestyle, socioeconomic differences such as education and income, low access to preventive health care services such as cholesterol screening, cardiac rehabilitation and a routine physical checkup [2, 35].The distribution of common MI risk factors (obesity, smoking) and mortality rate were considerably high at rural areas compared to urban areas in USA. The smoking and obesity rates in US rural are 29.6 and 39.6% compared to 24.2 and 33.4% in the urban USA population respectively [36, 37]. A higher AAIRMI in rural areas may be attributed to the difference in quality of life and lifestyle factors between rural and urban communities or it may arise from systematic differences between rural and urban cultures [38]. However, more data and further research are required to identify main reasons that are contributing to the difference in AAIRMI in rural and urban areas of Zanjan province.

Spatial analyses in this study revealed that AAIRMI patterns changes during 2014–2018. For example, the spatial distribution of hot spots was shifted the south from the center and the spatial distribution of cold spots was shifted the northeast from the southeast during 2014–2018. Addressing the place-based pattern of incidence and identifying the spatial and temporal patterns of AAIRMI is very important for policy planning at local level. The allocation of limited health resources should be focused on high-priority areas with the greatest risk of the incidence of MI. Because many patients die before even reaching the hospital, the key intervention is to educate the patient on symptoms and early arrival to the emergency department in the rural region. The results of this study provide ground work for future studies to explore risk factors of MI among high-risk areas in Zanjan province, Iran. Future research is needed to explore if there are any correlation between environmental risk factors like climate, humidity, temperature, air pollution, lifestyles and the incidence of MI in high risk areas of Zanjan province to identify the main risk factors and reduce their impact on the incidence of MI in rural and remote areas.

## Conclusion

This study provides an overview on the spatial distribution of the incidence of MI in Zanjan province, Iran, during 2014–2018. There are various causes of the incidence of MI including genetic, lifestyle, socioeconomic and environmental factors, which make it difficult to identify the main reasons of this public health concern. Our finding resealed that the AAIRMI was higher in men compared to the national average. Recent use of GIS to analysis or estimate spatial inequalities, highlighted many health problems which were found to be related to spatial differences or spatial inequalities. At conclusion, according to the 25 by 25 World Health Organization’s target [39], achieving a 25% global reduction in cardiovascular mortality by 2025, and whereas MI is a leading cause of death, the results of present study can help public health authorities to develop more effective prevention campaigns to reduce The incidence of MI across rural and remote areas. Further, spatial analysis will help policy maker to design prevention interventions for those area where the risk of MI is greater and reduce the burden for health system.

### Limitation

This study had three limitations. First, we collected and analyzed patients’ data from public hospitals in Zanjan and did not access to private hospital MI data. Therefore, it may not represent the overall MI patients in Zanjan province, Iran. The second limitation is the use of population census data as the dominant method for calculating the incidence rate of MI and age-adjusted incidence rate of MI. The population census is conducted every 5 years in Iran, and there is no annual population census data for each year. Therefore, we used national population census data in 2016 and estimated the population of other years by population annual growing rate, which reported annually by Statistical center of Iran. Third, HIS was implemented in some hospitals since 2014 and there was not complete data of The incidence of MI before 2014 in these hospitals, therefore, we only used patient data which were registered in HIS databases over a five-year period (from March 21, 2014 to March 21, 2019).

## Supplementary Information

**Additional file 1.**

## Data Availability

The datasets are available from the corresponding author on reasonable request.
