# Supplementary material for: Spatial and temporal analysis of myocardial infarction incidence in Zanjan province, Iran
Source: BMC Public Health. 2021 Sep 14;21:1667. doi: 10.1186/s12889-021-11695-8 (PMC8438974; doi:10.1186/s12889-021-11695-8)
Supplement: Supplementary file 1 — Additional file 1. [file 12889_2021_11695_MOESM1_ESM.docx]

**Additional file 1**

Global Moran’s I statistics for spatial autocorrelation is given as:

$$I=\frac{n\sum_{i=1}^{n} \sum_{j=1}^{n} W_{i,j}{(X_{i}-\mu)}^{2}}{\sum_{i=1}^{n} \sum_{j=1}^{n} W_{i,j}\sum_{i=1}^{n} {(X_{i}-\mu)}^{2}}$$

Where *n* was the total number of rural district,$X_{i}$ is AAIRMI of *i*’th rural district, $\mu$ was the mean of AAIRMIs and W*i,j* was the spatial weight between *i*’th rural district and *j*’th rural district.(21)

Getis-Ord G statistic is given as:

$$G_{i}=\frac{\sum_{j=1}^{n} W_{i,j}X_{j}-\mu\sum_{j=1}^{n} W_{i,j}}{S\sqrt{\frac{\left[ n\sum_{j=1}^{n} w_{i,j}^{2}- \left( \sum_{j=1}^{n} w_{i,j} \right)^{2} \right]}{n-1}}}$$

Where *X_j_* was AAIRMI for j rural district, W*i,j* was the spatial weight between *i*’th rural district and *j*’th rural district and n was the total number of rural district in this study.(24)

Anselin local Moran’s I is given as:

$$I_{i}=\frac{X_{i}-\mu}{\frac{\sum_{j=1,j\neq i}^{n} W_{i,j} \left( X_{j}-\mu\right)}{n-1}}\sum_{j=1,j\neq i}^{n} W_{i,j} (X_{j}-\mu)$$

Where *X_j_* was AAIRMI for *j*’th rural district, W*i,j* was the spatial weight between *i*’th rural district and *j*’th rural district and n was the total number of rural district in this study.(33)
